# Structural Characterization of Phosphorous Slag Regarding Occurrence State of Phosphorus in Dicalcium Silicate

**DOI:** 10.3390/ma15217450

**Published:** 2022-10-24

**Authors:** Yu Wang, Na Zhang, Huiteng Xiao, Jihan Zhao, Yihe Zhang, Xiaoming Liu

**Affiliations:** 1 Beijing Key Laboratory of Materials Utilization of Nonmetallic Minerals and Solid Wastes, National Laboratory of Mineral Materials, School of Materials Science and Technology, China University of Geosciences Beijing, Beijing 100083, China; 2 School of Metallurgical and Ecological Engineering, University of Science and Technology Beijing, Beijing 100083, China

**Keywords:** phosphorous slag, vitreous structure, solid solution, nuclear magnetic resonance, electron microprobe analysis

## Abstract

Phosphorous slag is a solid waste generated in the process of yellow phosphorus production. In order to deeply understand the structural and cementitious characteristics of phosphorous slag, comprehensive characterizations, including X-ray fluorescence spectrometry, X-ray diffraction, thermogravimetry, Fourier transform infrared spectrometry, Raman, scanning electron microscope, and inductively coupled plasma mass spectrometry were adopted to investigate the composition, thermal stability, microstructure, and cementitious activity of phosphorous slag. In addition, scanning electron microscope with energy dispersive X-ray spectroscopy, electron microprobe analysis, and solid-state nuclear magnetic resonance techniques were used to analyze the occurrence state of P in phosphorous slag. The results show that phosphorous slag is mostly vitreous with good thermal stability. Its chemical composition mainly comprises 43.85 wt % CaO, 35.87 wt % SiO_2,_ and 5.57 wt % Al_2_O_3_, which is similar to that of blast furnace slag, but it presents lower cementitious activity than blast furnace slag. P is uniformly distributed in the phosphorous slag with P_2_O_5_ content of 3.75 wt %. The distribution pattern of P is extremely similar to that of Si. P is mainly existing in orthophosphate of 3CaO·P_2_O_5_, which forms solid solution with dicalcium silicate (2CaO·SiO_2_). This work specifically clarifies the occurrence state of P in dicalcium silicate within the phosphorous slag. It is theoretically helpful to solve the retarding problem of phosphorous slag in cement and concrete.

## 1. Introduction

China is a major producer of yellow phosphorus, and phosphorous slag is a by-product of yellow phosphorus production [1]. In 2018, China produced 954,616 tons of yellow phosphorus, accounting for more than 80% of global production, and ranking first in the world. At present, there are more than 300 kinds of inorganic phosphorous chemical products using phosphorus as raw materials. The use of inorganic phosphorus can further produce organic phosphorus compounds. There are more than 10,000 inorganic phosphate and organic phosphorus compounds in the world [2,3]. At the same time, producing 1 ton of yellow phosphorus is accompanied by discharging 8–10 tons of phosphorous slag. It is estimated that 6 million tons of phosphorous slag is discharged annually in China [4,5,6], and the vast majority of phosphorous slag is piled up waiting for treatment, which not only occupies a large area of land but also causes the dissolution of fluorine and phosphorus after leaching by rain, polluting land and water resources and affecting plant growth and human health [7,8]. If phosphorous slag can be effectively recycled and utilized, it can bring huge social and economic benefits and provide strong support for the green development of industrial solid waste. However, phosphorous slag has a low utilization rate nowadays because of its physical and chemical properties [3]. The utilization rate of phosphorous slag is less than 30% in China [8].

The sustainable development of China’s yellow phosphorus industry is based on the in-depth development of block ore into a furnace, efficient utilization of ore, and deep processing techniques [9,10]. Due to the differences in the yellow phosphorus production process, the composition of phosphorous slag samples varies significantly with the change of collection points. The two main chemical components in the phosphorous slag are CaO and SiO_2_. Their total mass fraction is generally about 80%, and the mass fraction ratio of CaO/SiO_2_ is often between 1.1 and 1.3 [11,12,13]. Besides, phosphorous slag also contains different contents of Al_2_O_3_, Fe_2_O_3_, K_2_O, Na_2_O, MgO, and other components. The phosphorus that has not entered the final product generally exists in the phosphorous slag, and the mass fraction of P_2_O_5_ in the phosphorous slag is between 1.5% and 3.5% [1,11,12,13].

Since the total content of CaO, SiO_2,_ and Al_2_O_3_ in the phosphorous slag in various regions mostly exceeds 80%, more and more investigations have been carried out to use phosphorous slag in construction and building materials. Hu et al. [14] reported that superfine phosphorous slag powder can improve the pore structure of concrete, which is helpful to enhance the resistances of carbonation and the chloride ion permeability of concrete at a late age. Moreover, the superfine phosphorous slag is beneficial to the late-age development of compressive and splitting tensile strengths of concrete. Peng et al. [15] found that the porosity of concrete with high content of phosphorous slag powder and silica fume is very low, which endows the concrete with excellent mechanical properties and durability. Zhang et al. [16] pointed out that phosphorous slag hinders the early strength development of cement, and the hindrance can be reduced by increasing the curing temperature. Besides, the late-age compressive strength of phosphorous slag mortar far exceeds the compressive strength of ordinary cement mortar. Moreover, different curing temperatures have significant effects on the compressive strength of phosphorous slag-based cement [17]. Li et al. [18] found that phosphorous slag prolongs the setting time of cement, and the retardation problem can be solved when using appropriate admixtures such as calcined gypsum, calcined alumstone, and sodium sulfate. In general, the setting time of phosphorous slag-doped cement continues to increase with the increasing amount of phosphorous slag [19], mainly due to the formation of hydroxyapatite. It is thought that the occurrence state of P in phosphorous slag is an important factor related to the retardation and hydration mechanism of phosphorous slag-based cementitious materials. Therefore, it is necessary to understand the structural characteristics and occurrence state of P in the phosphorous slag.

The purpose of this work is to investigate the structural characteristics of phosphorous slag through X-ray diffraction (XRD), Fourier transform infrared spectrometry (FT-IR), Raman, thermogravimetry (TG), and scanning electron microscope (SEM) characterizations. The cementitious activity of phosphorous slag was evaluated by comparing it with that of blast furnace slag. In addition, scanning electron microscopy with energy dispersive X-ray spectroscopy (SEM-EDS), electron microprobe analysis (EMPA), and solid-state nuclear magnetic resonance (NMR) techniques were used to deeply analyze the occurrence state of P in phosphorous slag. This study lays a foundation for further research on the comprehensive utilization of phosphorous slag in cement and concrete. It helps to understand and explain the retarding phenomenon of phosphorous slag in the Portland cement blends and propose appropriate solutions for the retarding issue of phosphorus slag in cementitious materials.

## 2. Material and Characterization Methods

The phosphorous slag used in this experiment comes from Guizhou Kailin Co. Ltd., Guiyang, China. The granulated phosphorous slag was milled into powder with particle size d(0.5) of 18.08 μm. 5 g phosphorous slag powder was taken for chemical composition analysis, which was measured by Shimadzu XRF-1800 series X-ray fluorescence spectrometer (Kyoto, Japan). In order to confirm that phosphorous slag has a vitreous structure, the powder sample was tested with CuKα1 radiation on Rigaku D/MAX-RB X-ray diffractometer (Tokyo, Japan), the experimental conditions of which were as follows: 40 kV, 100 mA, scanning speed of 8°/min, scanning range of 5°–70°. Subsequently, we analyzed infrared spectrum of the powder sample, and the results were recorded by Renishaw inVia infrared spectrometer (Wotton-under-Edge, UK) using the KBr pellet technique. The specific steps were as follows: evenly mix 5 mg powder samples and 200 mg KBr, and then transfer them to a tablet press to make transparent slices for IR test. Simultaneously, 10 mg powder samples of phosphorous slag were tested by Renishaw inVia micro-Raman spectrometer (Britain) with an excitation wavelength of 785 nm. Furthermore, the thermal stability of phosphorous slag was tested by a simultaneous thermal analyzer (NETZSCH STA 449 F3, Selb, Germany), in which the powder sample was heated from 38 ℃ to 1000 °C at a heating rate of 10 °C/min in a stripping gas of dry N_2_. 

Scanning electron microscopy (SEM) combined with energy dispersive X-ray spectroscopy (EDS) was carried out by using Hitachi SU-8020 instrument (Chiyoda, TokyoJapan) under an operating voltage of 20 kV and current of 10 mA to study the microscopic morphology and element composition of the phosphorous slag. It should be noted that the raw granulated phosphorous slag was stuck on a conductive adhesive, and then the sample was treated with golden sputtering before SEM observation. 

In order to specifically analyze the occurrence state of P in the raw granulated phosphorous slag, JEOL JXA-8230 electron microprobe (Tokyo, Japan) was adopted to study the element distribution, which was operating at an accelerating voltage of 15 kV and a probe current of 10^−8^ A. Moreover, the powder sample of phosphorous slag was analyzed by Agilent 600 M solid-state nuclear magnetic resonance instrument (Santa Clara, CA, USA) to obtain the NMR spectra of ^27^Al, ^29^Si, and ^31^P. It was operating at 78.2 MHz for the ^27^Al resonance frequency, 59.6 MHz for the ^29^Si resonance frequency, and 11.3 MHz for the ^31^P resonance frequency. 

Alkaline dissolution method was used to compare the cementitious activity of phosphorous slag and blast furnace slag. The blast furnace slag powder was obtained from Tanglong Building Materials Co., Ltd., Tangshan, China. The procedure of alkaline dissolution experiment was performed as follows: taking 1 g powder sample into 50 mL NaOH (1 mol/L) solution and sealing it in a curing box at 20 °C. After 72 h, the solution was filtered, and the filtrate was sealed as samples. The concentration of Si, Al, and P in the filtrate was subsequently tested by an inductively coupled plasma mass spectrometer (Agilent 7500 ICP-MS, Santa Clara, CA, USA). In the above characterization tests, three times of replicate analyses were carried out to obtain the representative results.

## 3. Results and Discussion

### 3.1. Chemical Composition and Mineralogical Characteristics of Phosphorous Slag

**Figure 1:**
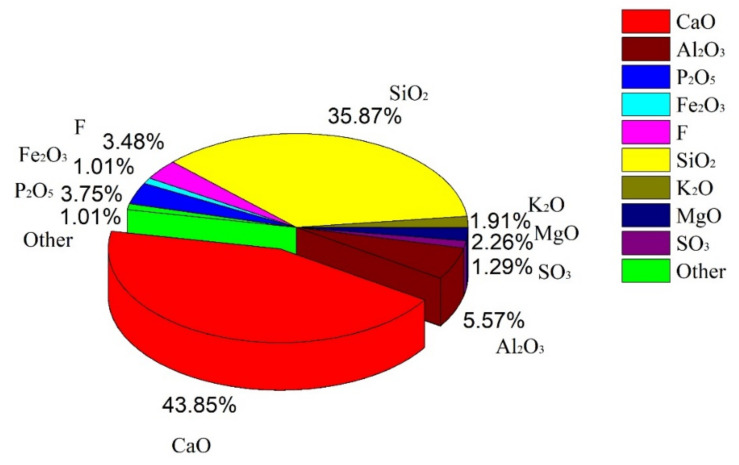
Chemical composition of phosphorous slag.

Figure 1 shows the mass fractions of the chemical components in the phosphorous slag. The total mass fraction of CaO and SiO_2_ is 79.72%, and the CaO/SiO_2_ mass ratio is 1.22, which are close to the values obtained by chemical analysis in other literature [20]. In addition to these two main components, the phosphorous slag contains a relatively high amount of Al_2_O_3_, P_2_O_5_, MgO, K_2_O, SO_3_, Fe_2_O_3_, and F-containing compounds. It is noticed that the content of P_2_O_5_ is about 3.75%, which means that the remaining P in the phosphorous slag still exceeds 1.5%. Through previous experiments, it is known that phosphorous slag cement has a significant retardation phenomenon [18]. If phosphorous slag is adopted into cement, appropriate measures must be taken to eliminate the influence of the P element, and hence it is essential to understand the occurrence state of P in the phosphorous slag. 

**Figure 2:**
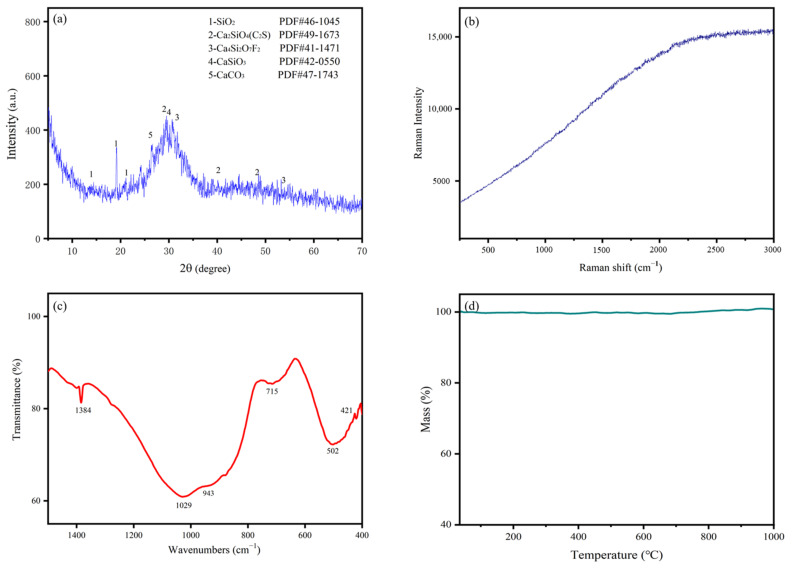
Basic characterization results of phosphorous slag: (**a**) XRD; (**b**) Raman; (**c**) FT-IR; (**d**) TG.

Figure 2 presents XRD, Raman, FT-IR, and TG characterization results. As shown in Figure 2a, the XRD pattern of the phosphorous slag has few sharp crystalline mineral peaks, but there is a main steamed bread-like peak at 2θ of 30°. It is confirmed that the phosphorous slag has a vitreous structure after rapid cooling treatment [7]. It also contains some crystal phases, such as cuspidine (Ca_4_Si_2_O_7_F_2_), wollastonite (CaSiO_3_) [8], dicalcium silicate (C_2_S), calcite (CaCO_3_), and quartz (SiO_2_). In addition to these crystal phases, most of the others are mainly composed in the vitreous structure, which accounts for 90% [21]. It is noticed from Figure 1 that 3.48% F is composed in the phosphorous slag. If the phosphorus slag contains soluble F, the soluble F will form a stable crystal structure with P to generate Ca_5_(PO_4_)_3_F, thereby significantly prolonging the setting time of the Portland cement [22]. However, according to the XRD pattern of the phosphorus slag shown in Figure 2a, the crystal phase of Ca_5_(PO_4_)_3_F has not been found. Instead, it is found that F is mainly occurring in the cuspidine (Ca_4_Si_2_O_7_F_2_), suggesting that F in this phosphorus slag is mainly insoluble, which would have little influence on the setting time of cementitious materials. 

Figure 2b shows a Raman spectrum without obvious peaks, demonstrating that the phosphorous slag mainly has a vitreous structure, which is consistent with the XRD analysis result. As Raman spectroscopy cannot directly show off molecular vibrations in vitreous structure, IR analysis was subsequently used. Both IR and Raman spectroscopy can provide information on molecular vibration frequencies. Although IR and Raman spectroscopy have different generation mechanisms, they can be complementary to each other, and more complete information on molecular vibrations can be obtained.

Figure 2c shows the FT-IR spectrum of the phosphorous slag. The absorption bands around 1384 cm^−1^ and 715 cm^−1^ are related to CO_3_^2−^ of calcite, corresponding to the anti-symmetric stretching vibration and bending vibration of C–O, respectively [23,24]. The absorption bands at 1029 cm^−1^ and 502 cm^−1^ are the common features of the infrared spectrum of silicate minerals, representing the antisymmetric stretching vibration of O–Si–O and the bending vibration of Si–O–X (X: tetrahedral Si or Al) [25]. The small shoulder at 421 cm^−1^ is corresponding to dicalcium silicate. It is interesting to note that an absorption band corresponding to the vibration of P–O appears around 943 cm^−1^. Because the P content is relatively low, the peak corresponding to the vibration of P–O is very weak. Combining the above characterization results, it can be found that the main phase components of phosphorous slag are dicalcium silicate, calcite, quartz, and vitreous silicate. 

Figure 2d shows the thermogravimetric (TG) curve of the phosphorous slag. It can be seen that the mass of phosphorous slag is almost unchanged from 38 °C to 1000 °C in a nitrogen atmosphere. The temperature for producing yellow phosphorus is 1350~1400 ℃ [21]. Because the phosphorous slag is produced at a very high temperature, it has good thermal stability. From the XRD and FTIR analysis results, calcite is found in the phosphorous slag sample. Generally speaking, calcite is decomposed around 750 ℃, which is accompanied by an appropriate mass loss in the TG curve. However, no clear mass loss is observed around 750 ℃ in Figure 2d, indicating that a small amount of CaCO_3_ is composed in the phosphorous slag sample. Considering that C_2_S is formed in the phosphorous slag during the high-temperature production process of yellow phosphorus, some C_2_S could react with water to form a small amount of hydrated calcium silicate in the wet milling of phosphorous slag, and finally, CaCO_3_ could be generated by the carbonation of the hydrated calcium silicate in the air. 

### 3.2. Micro-Morphology of Phosphorous Slag

**Figure 3:**
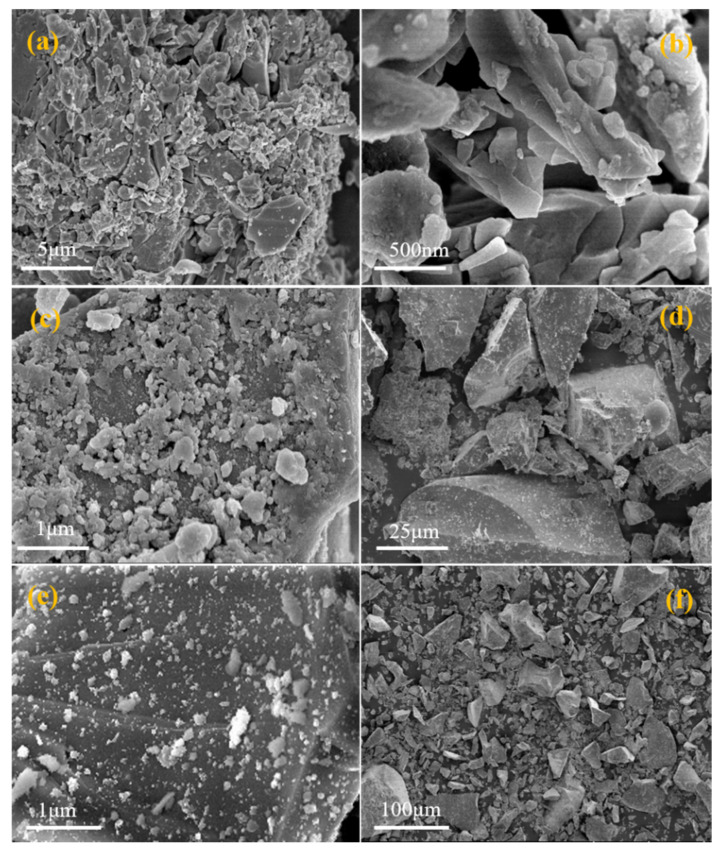
SEM morphology of phosphorous slag: (**a**–**f**) typical SEM images of phosphorous slag under different magnifications.

Through the XRD and Raman analysis results, we know that phosphorous slag has a vitreous structure. In order to further understand the structural characteristics of phosphorous slag, we need to observe its surface microstructure. Hence, the phosphorous slag was analyzed by scanning electron microscope (SEM) at different magnifications, the results of which are presented in Figure 3. From the SEM images, it can be seen that the phosphorous slag is mainly composed of irregular particles with rough surfaces and different sizes, and the distribution is disordered in Figure 3f. The surface of most phosphorous slag particles has no obvious pore structure, and the surface is relatively complete in Figure 3b,c, while the surface of a few particles is relatively broken and seems to be composed of a large number of small particles in Figure 3a,d. When an ordinary particle with a relatively complete surface is magnified 20,000 times in Figure 3e, it can be found that the particle is completely individual, and the surface is similar to an ordinary rock covered with a large number of irregularly impurities whose diameter is less than 1μm.

### 3.3. Occurrence State of P in Phosphorous Slag

#### 3.3.1. SEM-EDS Analysis

**Figure 4:**
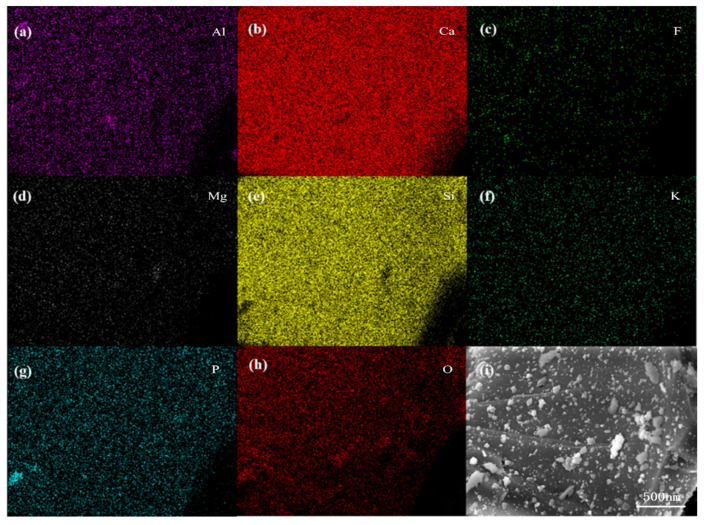
SEM-EDS elemental mappings of phosphorous slag: (**a**) Al; (**b**) Ca; (**c**) F; (**d**) Mg; (**e**) Si; (**f**) K; (**g**) P; (**h**) O; (**i**) scan position.

After analyzing the SEM images, we can only know the surface morphology of phosphorous slag. In order to obtain information about the main element distribution, Figure 4 displays SEM-EDS elemental mappings of Al, Ca, F, Mg, Si, K, P, and O in the phosphorous slag. It is noticed that these elements are evenly distributed in the phosphorous slag, and the distribution pattern of P is extremely similar to that of Si. 

#### 3.3.2. EMPA Analysis

**Figure 5:**
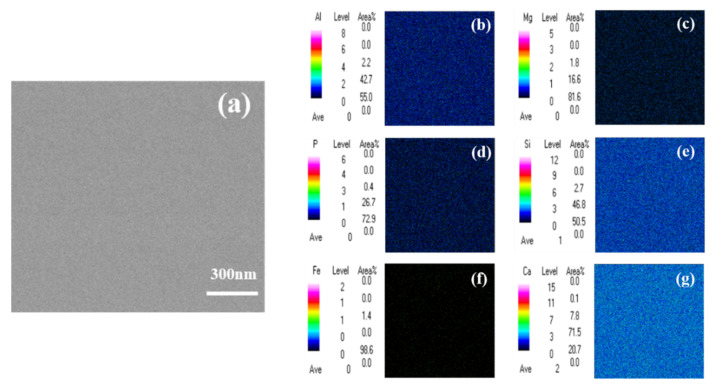
EMPA elemental mappings of phosphorous slag: (**a**) backscattered electron image; (**b**) Al; (**c**) Mg; (**d**) P; (**e**) Si; (**f**) Fe; (**g**) Ca.

It is impossible to accurately obtain the content of some trace elements only using SEM-EDS. On this basis, EMPA is especially suitable for the analysis of the chemical composition of the micro-area in the sample [26]. It can be used to study the distribution of the main elements and trace elements in the phosphorous slag. Figure 5 shows the EMPA mapping of P, Mg, Ca, Al, Si, and Fe in the phosphorous slag. It is known that the elements of P, Mg, Ca, Al, and Si are uniformly distributed, as seen in Figure 5, which is in accordance with the above SEM-EDS analysis result.

**Figure 6:**
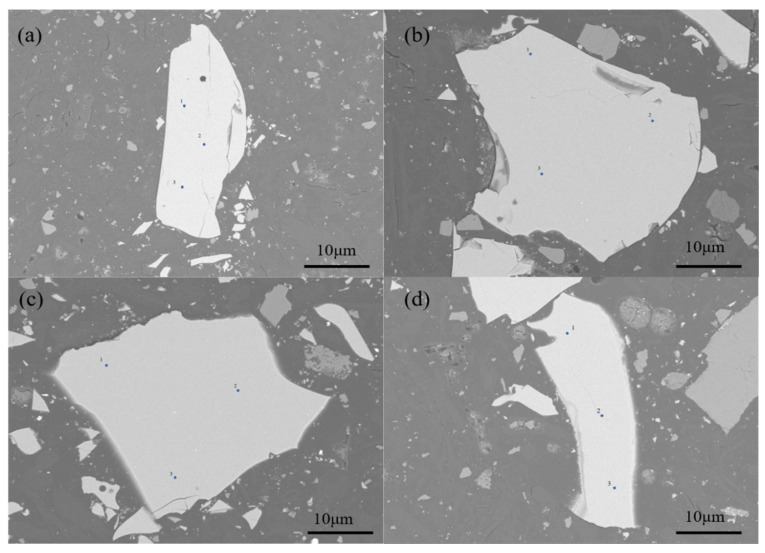
Backscattered electron image of phosphorous slag: (**a**–**d**) represents different positions of phosphorous slag respectively.

Figure 6 shows the selected points of EMPA quantitative analysis, and the chemical composition analysis results of each point are shown in Table 1. We can obtain that the main chemical composition of phosphorous slag measured by using EMPA is basically similar to the XRF analysis result. Besides, it contains a small amount of Na_2_O and FeO, which ranges from 0.31–0.38 wt % and 0.04–0.28 wt %, respectively. It can be found that F, with a mass fraction of at least 3% measured by XRF, did not appear in the EMPA analysis result. Theoretically, the EMPA method can be used to determine elements with atomic numbers greater than 3, but the quantitative results are only better for elements with atomic numbers greater than 10, while the quantitative results are not ideal for light elements with atomic numbers less than or equal to 10 [27]. As F has the lowest relative atomic number and atomic mass among the major elements contained in the phosphorous slag, F exceeds the measuring range of this electron probe microanalysis instrument [28]. 

It can be seen from Table 1 that the P_2_O_5_ content in the selected phosphorous slag samples is in a range of 3.02–4.15 wt %. Moreover, high contents of CaO and SiO_2_ are contained in the selected dots with a mole ratio of 1.17–1.27 correspondingly. Combining the SEM-EDS elemental mappings and the EMPA results, it is confirmed that P is uniformly distributed in the phosphorous slag, and it is mainly occurring in the Si-containing phase [29].

#### 3.3.3. NMR Analysis

Solid-state nuclear magnetic resonance mainly measures the absorption of radiation (4~600 MHz) by atomic nuclei, and it is an efficient method to investigate the structure of lower-crystallinity and amorphous materials. Considering that the P element in the phosphorous slag is uniformly distributed, and Al can replace part of Si in the [SiO_4_] tetrahedron to form an aluminosilicate structure, it is necessary to perform NMR analysis on ^27^Al, ^29^Si, and ^31^P to analyze the coordination structure of these elements in the phosphorous slag.

**Figure 7:**
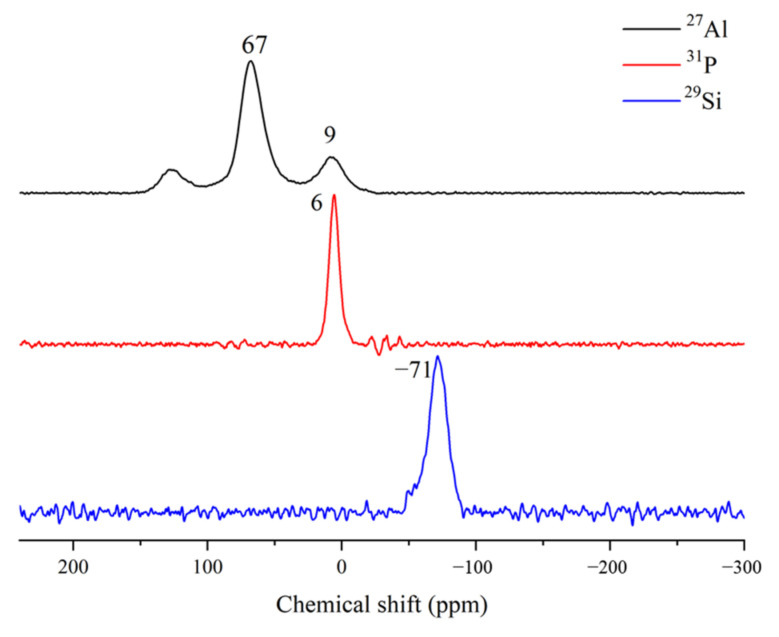
NMR spectra of ^27^Al, ^29^Si, and ^31^P.

Figure 7 shows the ^27^Al, ^29^Si, and ^31^P NMR spectra of phosphorous slag. There are two main resonance peaks of ^27^Al for the phosphorous slag. The resonance at a chemical shift of 9 ppm corresponds to Al^VI^, which is caused by octahedrally coordinated Al ([AlO_6_]) [30]. As the NMR signals of tetrahedrally coordinated Al occur between 55 and 80 ppm, the resonance at a chemical shift of 67 ppm corresponds to Al^IV^, which is generally related to cementitious activity [31]. It can be seen from Figure 7 that the peak intensity and relative peak area of Al^IV^ are larger than those of Al^VI^. It is thought that the phosphorous slag has potential cementitious activity.

The coordination structure of Si in silicates can be generally divided into SiQ^0^, SiQ^1^, SiQ^2^, SiQ^3^, and SiQ^4^ units according to the coordination number of bridging oxygen around Si in the [SiO_4_] tetrahedron. One major environment can be observed from the ^29^Si NMR spectrum of phosphorous slag. The position of −71 ppm is associated with the SiQ^0^ unit (orthosilicates) [32]. It is usually difficult to detect the amorphous phase by XRD technique, but it can be determined by NMR analysis. Thus, combining the ^29^Si NMR, XRD, and FT-IR analysis results of phosphorous slag, it is concluded that most of the siliceous substances exist in the form of SiQ^0^ unit, which is mainly arising from the dicalcium silicate in the phosphorous slag [32].

In the ^31^P NMR spectra of phosphorous slag, the resonance at a chemical shift of 6 ppm corresponds to the phosphorus environment of Q^0^ generated by orthophosphate groups [33,34]. Considering that the content of SiO_2_ in the phosphorous slag is much higher than that of P_2_O_5_ and Al_2_O_3_, dicalcium silicate is mainly formed during the cooling process of phosphorous slag. Based on the Q^0^ structure of P and Si, it is thought that in the phosphorous slag, P is mainly occurring in the dicalcium silicate phase as the form of orthophosphate. 

**Figure 8:**
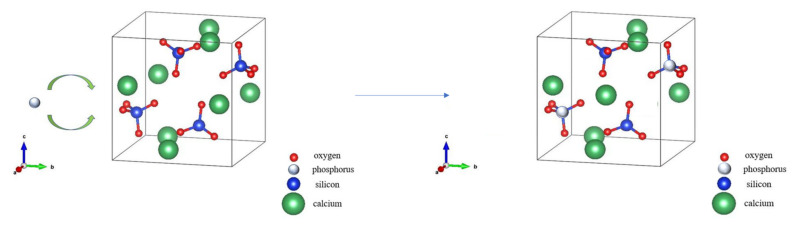
Schematic diagram of P participating in the structure of dicalcium silicate (C_2_S) in the phosphorous slag.

Wang et al. [35] investigated the existing form of P in the CaO–SiO_2_–Fe_n_O–P_2_O_5_ slag with different P content, in which the CaO-SiO_2_–Fe_n_O–P_2_O_5_ slag was prepared via heating to 1500 °C for 10 min, and then cooling to 1400 °C in the furnace, and finally cooling to the room temperature in the air. They found that P was concentrated in the solid solution of dicalcium silicate (2CaO·SiO_2_) and tricalcium phosphate (3CaO·P_2_O_5_) in the prepared high-phosphorus slag with a P_2_O_5_ content of 6% and 10%. With the increase of P_2_O_5_ content, it is beneficial to the enrichment and precipitation of the solid solution of 2CaO·SiO_2_ and 3CaO·P_2_O_5_. Herein, the phosphorous slag used in this work is derived from the production of yellow phosphorous at a high temperature of 1350–1400 °C. The residual P_2_O_5_ from the phosphate ore can quickly participate in the structure of 2CaO·SiO_2_ (as shown in Figure 8) during the cooling process, and it promotes the diffusion of CaO into 2CaO·SiO_2_ simultaneously, resulting in the solid solution of 2CaO·SiO_2_ and 3CaO·P_2_O_5_ [36]. This phenomenon has also been found by some researchers in the CaO-SiO_2_-P_2_O_5_ phase diagram [37,38]. In the molten phosphorus slag, Si is occurring in the form of [SiO_4_]^4−^, and P is existing in the form of [PO_4_]^3−^. Because the ionic radius of P (0.035 nm) is close to that of Si (0.039 nm), P atoms can substitute Si atoms in the crystal lattice of C_2_S to form the solid solution. The NMR analysis results obtained from Figure 7 show that the coordination structure of P and Si is Q^0^ in the phosphorous slag, which firmly verifies the solid solution of 3CaO·P_2_O_5_ and 2CaO·SiO_2_. 

### 3.4. Cementitious Activity of Phosphorous Slag

**Figure 9:**
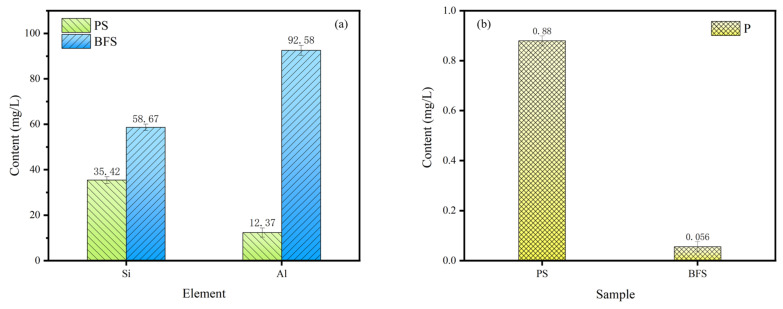
Dissolved content of Si, Al, and P from phosphorous slag (PS) and blast furnace slag (BFS) in 1 mol/L NaOH solution: (**a**) Si and Al, (**b**) P.

The cementitious activity of solid waste is usually evaluated by the content of active Si and Al dissolved in an alkaline environment. Considering that phosphorous slag mainly has a vitreous structure, and its main chemical composition is similar to that of blast furnace slag [39], the cementitious activity of phosphorous slag is compared with blast furnace slag. The dissolved contents of active Si and Al in 1 mol/L NaOH solution for phosphorous slag and blast furnace slag are presented in Figure 9. It can be seen that the dissolved contents of active Si and Al in the phosphorous slag are significantly lower than those of blast furnace slag. It is known that the content of SiO_2_ in the chemical composition of phosphorous slag and blast furnace slag is very close, which is around 30–35 wt%. However, the dissolved content of active Si in the phosphorous slag is 39.6% lower than that in the blast furnace slag. The content of Al_2_O_3_ in the blast furnace slag is 3 times of the Al_2_O_3_ content in the phosphorous slag, but the dissolved content of active Al in the blast furnace slag is 7.5 times that of the active Al content in the phosphorous slag. This disparity indicates that the cementitious activity of phosphorous slag is inferior to that of blast furnace slag. Figure 9 also displays the dissolved content of P in 1 mol/L NaOH solution for phosphorous slag and blast furnace slag. It is shocking that the dissolved concentration of P in the phosphorous slag is 15 times higher than that of the blast furnace slag. The active phosphorus leads to a serious retarding effect when the phosphorous slag is added to Portland cement due to the fact that hydroxyapatite is formed and precipitates on the cement particles, which hinders the hydration process [40]. The above analysis firmly indicates that improving the cementitious property of phosphorous slag and simultaneously preventing the generation of hydroxyapatite in the hydration process is a key point to promote the large-scale utilization of phosphorous slag in cement and concrete.

## 4. Conclusions

The purpose of this work is to understand the structural characteristics and occurrence state of P in phosphorous slag, which is helpful to guide the effective utilization of phosphorous slag in cement and concrete. The main conclusions are drawn as follows.

(1) The chemical composition of phosphorous slag mainly comprises 43.85% CaO, 35.87% SiO_2_, 5.57% Al_2_O_3_, 3.75% P_2_O_5_, 2.26% MgO, and 3.48% F-containing compounds. Phosphorous slag has a vitreous structure, which endows it with good thermal stability due to the high production temperature of yellow phosphorus, and it can be used as a supplementary cementitious material. The main phase components of phosphorous slag are dicalcium silicate, calcite, quartz, and vitreous silicate.
(2) SEM observation shows that phosphorous slag is mainly composed of irregular particles with rough surfaces and different sizes, and the distribution is disordered. It has no obvious pore structure, and the surface is relatively complete, which is similar to the surface of ordinary rock.
(3) Tetrahedrally coordinated Al is mostly existing in the phosphorous slag, which is beneficial to the cementitious activity. P is evenly distributed in the phosphorous slag, and its distribution pattern is extremely similar to that of Si. The coordination structure of P and Si is Q^0^ in the phosphorous slag, supporting that P is occurring in the solid solution of 3CaO·P_2_O_5_ and 2CaO·SiO_2_.
(4) The occurrence state of P in dicalcium silicate within the phosphorous slag is clarified. The phosphorous slag used in this work is derived from the production of yellow phosphorous at a high temperature of 1350–1400 °C. The residual P_2_O_5_ from the phosphate ore can quickly participate in the structure of 2CaO·SiO_2_ during the cooling process, and it promotes the diffusion of CaO into 2CaO·SiO_2_ simultaneously, resulting in the solid solution of 2CaO·SiO_2_ and 3CaO·P_2_O_5_ in the phosphorous slag.
(5) The cementitious activity of phosphorous slag is inferior to that of blast furnace slag. The dissolved content of P in 1 mol/L NaOH for the phosphorous slag is 15 times higher than that for the blast furnace slag. Improving the cementitious property of phosphorous slag and simultaneously solidifying the active phosphorus to prevent the generation of hydroxyapatite in the hydration process is a key point to promoting the large-scale utilization of phosphorous slag in cement and concrete. Hopefully, we are developing a new kind of silica-alumina-based cementitious material composed of phosphorous slag, which can solve the retarding issue of phosphorous slag. It can promote the extensive utilization of phosphorous slag in construction and building materials with considerable ecological and economic benefits. This is an important and interesting subject to be investigated and reported on in our future work.

## Figures and Tables

**Table 1:** EMPA quantitative analysis results of marked dots in Figure 6.

Point	Main Components (wt %)								
	Na_2_O	P_2_O_5_	Al_2_O_3_	MgO	CaO	K_2_O	SiO_2_	FeO	SO_3_
a-1	0.309	3.129	5.692	2.304	43.843	1.687	39.876	0.060	1.680
a-2	0.307	3.239	5.778	2.336	43.900	1.649	40.168	0.169	1.729
a-3	0.365	3.167	5.759	2.287	43.524	1.607	39.896	0.144	1.522
b-1	0.363	3.136	5.655	2.272	44.693	1.468	38.431	0.281	1.934
b-2	0.341	3.019	5.894	2.184	44.465	1.475	38.517	0.041	1.947
b-3	0.367	3.049	5.684	2.195	44.590	1.448	37.953	0.039	1.830
c-1	0.374	3.566	5.594	2.234	45.093	1.500	38.396	0.186	1.943
c-2	0.347	3.505	5.639	2.165	44.922	1.555	37.895	0.154	1.937
c-3	0.380	3.610	5.637	2.249	44.689	1.509	37.607	0.069	1.935
d-1	0.384	3.980	5.671	2.177	42.790	1.832	38.542	0.045	1.828
d-2	0.318	4.146	7.712	2.223	43.241	1.850	38.403	0.105	1.933
d-3	0.380	3.982	5.719	2.182	43.281	1.811	38.353	0.060	1.967

## Data Availability

Data will be made available on request.
